# O.4.1-8 Association of sit-to-stand capacity and free-living performance using thigh-worn accelerometers among 60-90-year-old adults

**DOI:** 10.1093/eurpub/ckad133.173

**Published:** 2023-09-11

**Authors:** Antti Löppönen, Christophe Delecluse, Kristin Suorsa, Laura Karavirta, Tuija Leskinen, Lien Meulemans, Erja Portegijs, Taija Finni, Taina Rantanen, Sari Stenholm, Timo Rantalainen, Evelien Van Roie

**Affiliations:** KU Leuven, Belgium; University of Jyväskylä, Finland; KU Leuven, Belgium; University of Turku, Turku University Hospital, Finland; University of Jyväskylä, Finland; University of Turku, Turku University Hospital, Finland; KU Leuven, Belgium; University of Groningen, Netherlands; antti.ej.lopponen@jyu.fi; University of Jyväskylä, Finland; University of Jyväskylä, Finland; University of Turku, Turku University Hospital, Finland; University of Jyväskylä, Finland; KU Leuven, Belgium

## Abstract

**Introduction:**

Five times sit-to-stand (STS) test is commonly used as a clinical assessment of lower-extremity functional ability, but its association with free-living performance has not been studied. Therefore, we investigated the association between laboratory-based STS capacity and free-living STS performance using accelerometry. The results were stratified according to age and functional ability groups.

**Methods:**

This cross-sectional study included 497 (63% women) participants aged 60–90 years from three independent studies. A thigh-worn tri-axial accelerometer was used to estimate angular velocity in maximal laboratory-based STS capacity and in free-living STS transitions over 3-7 days of continuous monitoring. Functional ability was assessed with Short Physical Performance Battery (SPPB).

**Results:**

Laboratory-based STS capacity was moderately associated with the free-living mean and maximal STS performance (r = 0.52 - 0.65, p < .01). Angular velocity was lower in older compared to younger and in low- versus high-functioning groups, both in capacity and free-living STS variables (all p < .05). Overall, angular velocity was higher in capacity compared to free-living STS performance. The STS reserve (test capacity – free-living maximal performance) was larger in younger and in high-functioning compared to older and low-functioning groups (all p < .05).

**Conclusion:**

Laboratory-based STS capacity and free-living performance were found to be associated. However, capacity and performance are not interchangeable, but rather provide complementary information. Older and low-functioning individuals seemed to perform free-living STS movements at a higher percentage of their maximal capacity compared to younger and high-functioning individuals. Therefore, we postulate that low capacity may limit free-living performance.

**Support/Funding Source:**

This study was supported by the Research Foundation Flanders, Belgium, the Academy of Finland, the Finnish Ministry of Education and Culture and an Advanced Grant from the European Research Council.

